# Data-augmented machine learning refines the effective-concentration estimate for eculizumab in complement-mediated diseases

**DOI:** 10.3389/fimmu.2026.1800803

**Published:** 2026-07-24

**Authors:** Francisco J. Fernández, Lucía Alfonso-González, Manuel Praga, Kaare Mikkelsen, M. Cristina Vega

**Affiliations:** 1 Dep. Molecular and Cellular Biosciences, Centro de Investigaciones Biológicas Margarita Salas (CIB-CSIC), Consejo Superior de Investigaciones Científicas, Madrid, Spain; 2 Abvance Biotech SL, Pharmacokinetics, Pharmacodynamics & Drug Metabolism (PPDM), Madrid, Spain; 3 Department of Medicine, Complutense University, Madrid, Spain; 4 Department of Electrical and Computer Engineering, Aarhus University, Aarhus, Denmark

**Keywords:** atypical hemolytic uremic syndrome (aHUS), complement factor C5, complement system, eculizumab (monoclonal antibody to C5), machine learning (ML), paroxysmal nocturnal hemoglobinuria (PNH), synthetic data augmentation

## Abstract

Eculizumab, a humanized monoclonal antibody targeting the complement protein C5, is highly efficient in paroxysmal nocturnal hemoglobinuria, atypical hemolytic uremic syndrome, generalized myasthenia gravis, and neuromyelitis optica spectrum disorder. However, recent reports have highlighted a subset of patients who show inadequate treatment response, prompting dose escalation or interval shortening. The serum concentration required for sustained inhibition of the complement lytic pathway remains uncertain, and the commonly cited 50–100 µg/ml range is not grounded in robust pharmacokinetic–pharmacodynamic data. Using publicly available clinical data digitized from four rare indications, we trained machine-learning supervised classifiers to predict complete C5 inhibition as a function of serum eculizumab concentration, identifying logistic regression on log-transformed concentration as the best and mechanistically appropriate model on real data. We then examined whether augmenting the real training data with high-fidelity synthetic data improves the resulting estimate. Although augmentation did not significantly change balanced accuracy, it reduced the confidence interval for the effective concentration by roughly 12-fold (a 92% reduction). The augmentation-stabilized estimate of the concentration associated with complete C5 inhibition was approximately 310.8–335.2 µg/ml (real-data-only estimate 338 µg/ml, 95% CI 275–454). These data-driven, hypothesis-generating results indicate that current maintenance targets aimed at near-complete C5 suppression may be substantially underestimated and that faithful synthetic augmentation can improve the precision of model-based pharmacological estimates in rare diseases; prospective validation in independent cohorts is required before any clinical application.

## Introduction

Eculizumab (Soliris^®^) is a humanized monoclonal antibody that binds complement protein C5, preventing its cleavage into C5a and C5b and thereby blocking formation of the terminal complement complex (1). It is approved by the FDA and EMA for several rare complement-mediated or complement-amplified disorders, including paroxysmal nocturnal hemoglobinuria (PNH; ORPHA: 67038), atypical hemolytic uremic syndrome (aHUS; ORPHA: 213653), generalized myasthenia gravis (gMG; ORPHA: 512), and neuromyelitis optica spectrum disorder (NMOSD; ORPHA: 71291) (2). Across these indications, eculizumab has demonstrated efficacy in reducing disease activity and improving patient outcomes (2). Its principal safety concern is increased susceptibility to invasive meningococcal disease, requiring vaccination and vigilance (3); hepatotoxicity and other long-term effects are increasingly reported and warrant systematic evaluation (4, 5).

Although eculizumab has transformed the treatment of these diseases, the exposure required to maintain complete inhibition of the terminal pathway remains incompletely defined. The conventional target range of approximately 50–100 µg/ml has become a practical reference point, but it was not derived from a large disease-stratified pharmacokinetic-pharmacodynamic (PK-PD) study and has therefore been called into question (1, 6). Clinical reports show that some patients have breakthrough complement activity or clinical relapse despite guideline-based treatment (7), whereas others accumulate high trough concentrations, particularly in pediatric or clinically complex settings (8, 9). This uncertainty is compounded by the exceptionally high cost of therapy, estimated at US$ 700,000 per patient annually. These observations create a therapeutic dilemma: insufficient exposure may permit residual C5 activation, while excessive exposure increases cost and may compound safety concerns in a treatment already associated with susceptibility to invasive meningococcal infection and other reported adverse effects. Together, these factors motivate more precise, data-grounded estimates of the effective maintenance concentration of therapeutic antibodies.

Rare diseases pose a structural challenge to conventional exposure-response modeling due to the fundamental problem of “small-*n*” datasets. Machine learning (ML) can be useful in this setting for deriving data-driven concentration estimates, with synthetic data generation to partially address inherent data scarcity by augmenting datasets that pool real and synthetic data (10–12). However, the application of ML methods to augmented datasets must be approached conservatively. Real clinical observations must remain the primary evidence, synthetic data must be screened before use, and augmented models must be evaluated on real observations rather than on synthetic samples. Rather than using synthetic data to prescribe a new clinical target, we use the real dataset to establish the benchmark exposure-response classifier and then test whether carefully screened synthetic augmentation improves model stability and specificity in a rare-disease pharmacodynamic problem. This approach may be important for complement-mediated rare disorders, where the scarcity of trial participants and the heterogeneity of disease symptoms make traditional data collection challenging for reliable pharmacological modeling.

In this study we extracted clinical data for eculizumab and antibody-free C5 concentrations from a handful of publicly available studies on PNH, aHUS, gMG, and NMOSD, and trained and tested ML classifiers on this small data set (*n* = 390 observations) to construct a transparent, reproducible classifier of complete C5 blockade from serum eculizumab concentration that could be applied across the various indications where eculizumab is the chosen therapy. We also aimed to determine whether augmentation improves performance when evaluated exclusively on real data. Our ML model predicts the C5 inhibition status from serum eculizumab concentration alone with remarkable precision and indicates that the target concentration should be significantly higher than the often-cited range of 50–100 μg/ml to achieve more complete inhibition of the complement system’s lytic pathway.

## Methods

### Clinical data sources and endpoint definition

Paired serum eculizumab and free (unbound) C5 measurements were compiled from clinical pharmacology and efficacy studies corresponding to trials NCT00844545, NCT00838513, NCT01997229, NCT01892345, NCT02946463, and NCT03056040. The final analysis dataset contained 390 observations (**Supplementary Table 1**). The binary pharmacodynamic endpoint was complete terminal pathway blockade, defined as free C5 ≤ 0.5 µg/ml. Observations above this value were labeled as not fully inhibited. This endpoint was selected because free C5 has been used as a direct pharmacodynamic marker of target engagement and because the original sources did not provide harmonized patient-level terminal pathway biomarkers such as CH50, AH50, or sC5b-9. The dataset is pooled across PNH, aHUS, gMG, and NMOSD. The available public material does not provide sufficient patient-level covariates for a reliable stratified model by indication, age, body weight, renal function, concomitant treatment, complement genotype, or sampling time relative to infusion. These variables are therefore not included as model features. The analysis should consequently be interpreted as an eculizumab exposure-free-C5 model, not as a disease-specific dosing model.

### Digitization and reliability analysis

Because tabular data were not consistently available, values were extracted from published figures with PlotDigitizer (https://plotdigitizer.com). PlotDigitizer has been validated for accurate numerical extraction, with a Pearson correlation coefficient of 0.982 between true and extracted values (13). To quantify the potential effect of this step, source figures were independently re-digitized, and the resulting x/y agreement was assessed. The repeated extraction yielded RMSE values of 1.25 and 0.68 for the x and y axes, respectively, and Pearson correlation coefficients of 0.9998 and 0.9992.

### Concentration-effect modeling and effective-concentration estimation

The dataset pairs each independent patient’s serum eculizumab concentration with a free C5 measurement at an arbitrary treatment time, so the conditional relationship free C5 = f([eculizumab]) describes the target pharmacology. We therefore modeled free C5 directly as a continuous function of serum eculizumab and defined the effective concentration as the serum eculizumab at which the fitted free-C5 curve crosses the inhibition cut-off. Observations with serum eculizumab > 0 were retained (*n* = 359 of 390) so that concentration could be placed on a log scale; free C5 was floored at 0.001 µg/ml for the log transform. A concentration-effect form was fitted on the log_10_ (free C5) scale by nonlinear least squares: a four-parameter log-logistic model (4PL) in log_10_ (concentration). Because a substantial fraction of free-C5 values lies near the assay limit of detection (LOD, 0.01 µg/ml), we additionally fitted a left-censored (Tobit) 4PL by maximum likelihood, treating observations ≤ LOD as left-censored rather than clipped, as a robustness check on the lower plateau. Goodness of fit was summarized by the coefficient of determination (R²) on the log_10_ scale.

The effective concentration was the serum eculizumab at which the fitted curve equaled the inhibition cut-off (free C5 = 0.5 µg/ml), located by Brent’s method (14, 15). Its uncertainty was quantified by a nonparametric bootstrap (1,000 resamples of the paired observations with per-replicate refitting), reported as the 95% confidence interval of the bootstrap distribution. To characterize the floor below which no dose can drive free C5, we report the lower plateau from both the clipped-OLS fits and the less-biased left-censored Tobit fit, and the baseline (upper plateau) (16). Finally, because the estimate depends on the chosen cut-off, we screened the effective concentration across free-C5 cut-offs from the plateau upward and anchored a “% of baseline” criterion (20% of baseline ≈ 80% free-C5 reduction). As a model-free check, we computed the empirical fraction of observations with free C5 ≤ 0.5 µg/ml within deciles of serum eculizumab concentration, with Wilson 95% confidence intervals. Analyses were performed using NumPy, SciPy, and pandas in Python 3 (17).

### Machine learning pipeline on real data

Observations were labeled Inhibition = True when free C5 ≤ 0.5 µg/ml (*n* = 87) and False otherwise (*n* = 303); this threshold is a previously validated indicator of complete inhibition (18). The predictor variable was serum eculizumab concentration.

Seven classifier families were evaluated: logistic regression (LR), random forest (RF), Gaussian naïve Bayes (GNB), k-nearest neighbors (KNN), support vector machine (SVM), gradient boosting classifier (GBC), and XGBoost. Models were built using scikit-learn (v1.8.0) (19) and XGBoost (v3.2.0) (20) in Python 3 (17). Five preprocessing strategies were considered where appropriate: raw concentration, standard scaling, robust scaling, log_10_ transformation, and log_10_ transformation followed by standard scaling. Allowed classifier-preprocessor combinations were defined *a priori* based on algorithm assumptions, yielding 31 combinations.

Model selection used 10-fold cross-validation with shuffling and a fixed random seed. Mean per-fold balanced accuracy (with per-fold scores retained for statistical testing) was the primary selection metric because the inhibited class was underrepresented (87/390; 22.3%). Accuracy, specificity, Matthews correlation coefficient (MCC), Cohen’s κ, ROC AUC, and average precision were reported as secondary metrics. Hyperparameters were kept at default values to avoid optimistic overfitting in a small dataset; formal tuning should be reserved for larger datasets and conducted with nested cross-validation. The best real-only pipeline was fixed before downstream holdout and augmentation analyses.

A stratified 80:20 train-test split was used for holdout evaluation. The training set contained 312 real observations, and the holdout set contained 78 real observations. Synthetic samples were never included in the holdout set used to estimate predictive performance.

### Synthetic data generation and fidelity screening

Five tabular generators were evaluated: SingleTablePreset, GaussianCopula, CTGAN, TVAE, all from the Synthetic Data Vault (SDV) library (v0.18) (21), and a transformer-based generator (MostlyAI; https://mostly.ai). Each was fitted to the full real dataset (*n* = 390) and used to produce synthetic data with a fixed random seed. Before any augmented training, synthetic datasets were screened for distributional fidelity to real serum eculizumab concentrations. Screening included descriptive statistics on the raw and log_10_ scales; two-sample Kolmogorov-Smirnov, Anderson-Darling, and Mann-Whitney tests at α = 0.05; Cohen’s *d* on log_10_-transformed values (negligible if |*d*| < 0.2); class balance comparison; visual diagnostics, including the kernel density estimate (KDE), empirical cumulative distribution function (ECDF), and Q-Q plots; and discriminator testing. Synthetic data fidelity was also assessed using a classifier two-sample test (C2ST) (22). Real (label 0) and synthetic (label 1) records were pooled, and a logistic-regression discriminator was trained to separate the two sources. Discriminability was quantified using balanced accuracy in stratified 10-fold cross-validation, with each held-out fold containing both real and synthetic records. Under the null hypothesis that the synthetic and real distributions coincide, the expected balanced accuracy is the chance level of 0.5; fidelity was therefore judged by proximity to 0.5, interpreted as low detectability of synthetic artifacts. We report the effect size as the absolute distance from chance, |balanced accuracy − 0.5|, and assess equivalence to chance with two one-sided tests (TOST) against a pre-specified margin of δ = [0.05]. A balanced accuracy that is statistically equivalent to 0.5 and does not significantly exceed it indicates that the discriminator cannot separate synthetic from real records, i.e., there are no detectable synthetic artifacts. In addition, a larger synthetic data set was created using MostlyAI with a sample size of *n* = 10,000 data points per binary class.

### Synthetic data augmentation and evaluation

The fixed real-data pipeline was retrained on real data concatenated with increasing amounts of MostlyAI synthetic data and evaluated on the same fixed real holdout (no synthetic samples in any performance estimate). Three controls established what augmentation contributes. First, a size-matched control, in which at each augmentation fraction (25%, 50%, 75%, and 100%), the real training set was subsampled without replacement to the same total *n*, isolating the informational benefit from sample-size effects. Second, a significance test consisting of a one-sided paired Wilcoxon signed-rank test on per-fold balanced accuracy compared the best real-only and best real-plus-synthetic configurations. Third, precision was assessed using bootstrap confidence intervals for the effective concentration, computed on pooled out-of-fold real predictions, and compared across augmentation levels. The same model family was held fixed across all scenarios so that any change is attributable to the data, not to a change of model.

We then investigated the effect of progressively increasing the synthetic-to-real ratios (0.5×, 1×, 2×, 4×, and 8×; ratio 0 = real only). All synthetic sampling was strictly without replacement. The deployed model was reused unchanged across all scenarios; no model or preprocessing reselection was permitted, so that performance differences are attributable to augmentation alone. Two complementary synthetic draws were used to reflect the two distinct estimands. For the performance assessment, synthetic points were drawn to ensure the combined real−plus−synthetic training set was class−balanced (the minority-inhibited class receiving the class deficit), thereby removing the cohort’s imbalance from classifier training. For the threshold estimate, synthetic points were instead drawn matched to the real class prevalence, so that the prior seen by the unweighted logistic fit, and hence the location of its P(inhibition) = 0.5 crossing, was preserved. Classifier performance was quantified on the 20% holdout and, in parallel, by 10−fold stratified out−of−fold cross−validation on the augmented training set, using balanced accuracy, accuracy, specificity, MCC, Cohen’s κ, ROC AUC, and average precision (area under the PR curve), with the inhibited class as positive.

The effective concentration was defined as the serum eculizumab concentration at which the unweighted (maximum−likelihood) logistic model predicts P(inhibition) = 0.5. LOD−substituted (zero) concentrations were excluded from the crossing fit but retained for classification. For each scenario, a 95% confidence interval on the crossing was obtained by a bootstrap (5000 replicates) in which only the real observations were resampled (at constant size), and the full synthetic block was appended unchanged in every replicate; resampling the pooled real−plus−synthetic set was deliberately avoided, as it would shrink the interval trivially with sample size. These threshold estimates are explicitly not converted into dosing recommendations because dose selection requires time-course PK, trough timing, body weight, renal function, disease activity, concomitant treatment, and safety-economic considerations that are not present in the public dataset.

## Results and discussion

### Data provenance and model transparency

**Figure 1:**
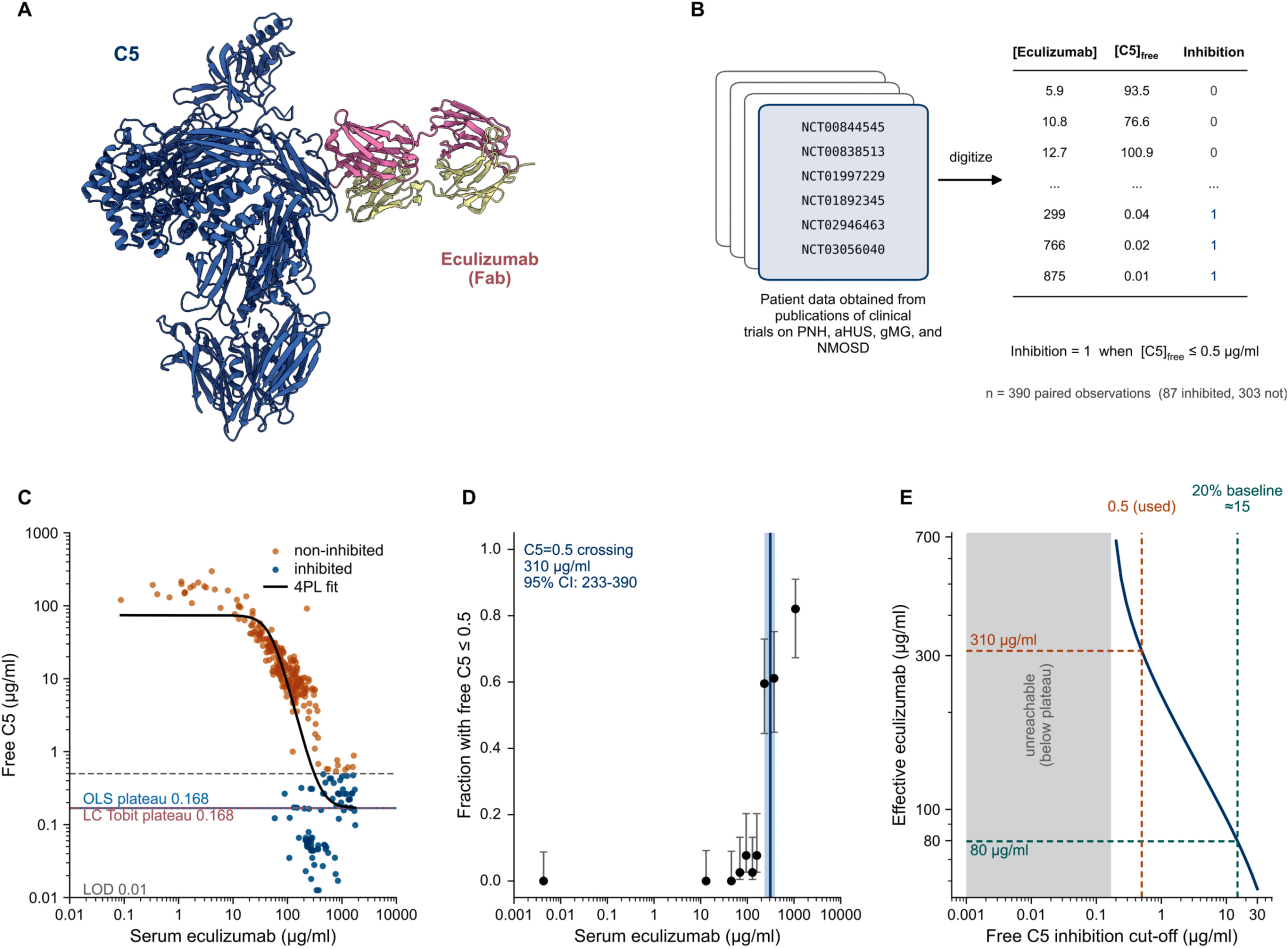
Concentration-effect relationship between serum eculizumab and free C5, and the resulting effective-concentration estimate. **(A)** Crystal structure of the C5-eculizumab (Fab) complex (PDB 5I5K): C5 (blue) bound by the eculizumab antigen-binding fragment (heavy and light chains in pink and yellow). **(B)** Data provenance and label coding. Paired serum eculizumab and free (unbound) C5 measurements were digitized from published figures of six clinical-trial reports (ClinicalTrials.gov identifiers shown). **(C)** Free C5 versus serum eculizumab (both log_10_ scale), colored by inhibition status. The solid black line is the 4PL fit; the dashed grey line marks the 0.5 µg/ml inhibition cut-off and the dotted grey line the assay limit of detection (LOD, 0.01 µg/ml). Horizontal lines indicate the lower plateau (free-C5 floor) estimated by clipped ordinary least squares (OLS, light blue) and by the left-censored Tobit fit (purple); both lie at 0.168 µg/ml. **(D)** Model-free corroboration. Points show the fraction of observations with free C5 ≤ 0.5 µg/ml within each decile of serum eculizumab (plotted at the within-decile geometric mean concentration; error bars are Wilson 95% confidence intervals). The vertical blue line and shaded band represent the fitted free-C5 = 0.5 µg/ml crossing and its 95% bootstrap confidence interval. **(E)** Sensitivity of the effective concentration to the chosen free-C5 cut-off. The blue curve is the serum eculizumab at which fitted free-C5 equals each cut-off; the grey region below the plateau is pharmacologically unreachable. Dashed lines mark the 0.5 µg/ml cut-off used (vermillion) and a “20% of baseline” anchor (~15 µg/ml, green).

We have reassessed the serum eculizumab concentration required to completely inhibit C5 activation in four rare complement-associated disorders: PNH, aHUS, gMG, and NMOSD. To this end, we collected publicly available clinical data from small multicenter studies across the four rare indications (**Figure 1B**; **Supplementary Table 1**). We first analyzed the data using a pharmacologically appropriate logistic regression model to determine the serum eculizumab concentration required to achieve ≤ 0.5 µg/ml of uncomplexed (free) C5, the threshold at which most studies consider full C5 inhibition to be achieved. We then trained supervised ML models to predict the likelihood of complete C5 inhibition as a function of the maintenance drug concentration (x), using the free C5 concentration (y) as the target. Patient data had to be obtained through careful digitization of publicly available sources using dedicated software tools, and the reliability of these data was explicitly assessed. Acknowledging the data scarcity inherent in rare diseases, we used synthetic data augmentation and evaluated its impact on model performance. Our study adheres to the TRIPOD-AI checklist (**Supplementary Table 2**).

### Pharmacokinetic modeling of eculizumab/free C5 data

Previous studies in patients with PNH, aHUS, gMG, or NMOSD receiving eculizumab have established a saturable (sigmoidal) concentration-effect relationship between serum eculizumab concentration and free C5, reflecting the progressive reduction in free C5 with increasing serum eculizumab concentration (and hence reduced C5 activation and hemolysis). We fitted the experimental data to a saturating 4-parameter logistic (4PL) regression model (R^2^ = 0.68) (**Figure 1C**). The fitted baseline (upper plateau) was 74 µg/ml (reported physiological concentration of C5 in healthy adults is approximately 75 µg/ml) (23), with a half-maximal concentration (EC_50_) of 49.6 µg/ml, and a lower plateau (free-C5 floor) of 0.168 µg/ml. This floor was robust to assay censoring: the clipped-OLS and left-censored Tobit estimates coincided at 0.168 µg/ml (**Figure 1C**), indicating that the plateau is a genuine feature of the concentration-effect relationship. The 0.5 µg/ml cut-off represents a stringent criterion, as it implies near-complete (~99%) suppression of free C5 relative to a baseline of ~74 µg/ml. This threshold has been reported to correspond to ≤ 20% hemolysis (18), indicating that minute amounts of free C5 can sustain a significant yet subclinical level of hemolysis.

Using the pre-specified inhibition cut-off (free-C5 = 0.5 µg/ml), the fitted 4PL dose-response gave an effective serum eculizumab concentration of 310 µg/ml (bootstrap 95% CI 233–390; **Figure 1C**, with the estimate and interval overlaid in **Figure 1D**). A model-free analysis was consistent: the empirical fraction of patients with free-C5 ≤ 0.5 µg/ml remained near zero up to ~100 µg/ml and crossed one-half within the bootstrap interval (**Figure 1D**), so the threshold is supported by the raw data independently of the parametric form. This effective concentration is significantly above the commonly cited 50–100 µg/ml maintenance range. At 50 µg/ml, serum eculizumab matches the EC_50_, thereby achieving a 50% free-C5 reduction, while ~80 µg/ml serum eculizumab accomplishes an 80% reduction in free-C5, which equals ~15 µg/ml (**Figure 1E**).

Taken together, these results indicate that achieving near-complete C5 suppression requires serum eculizumab above the commonly cited 50–100 µg/ml maintenance range.

### A logistic ML dose-response model performs best on real data

**Figure 2:**
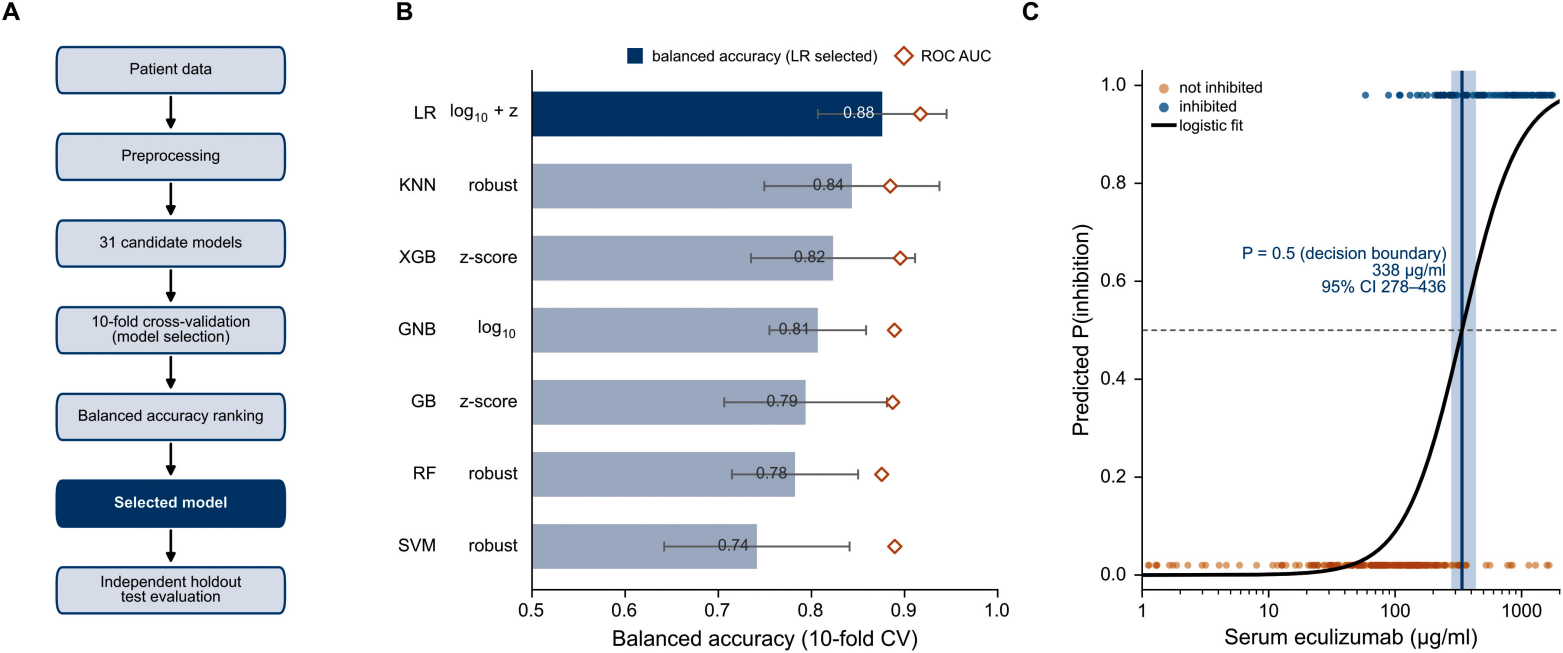
Classifier-based estimation of the serum eculizumab concentration associated with complement (free C5) inhibition. **(A)** Analysis pipeline. Pooled patient observations (n = 390 paired serum eculizumab/free C5 measurements) were preprocessed and passed to a pre-specified screen of 31 preprocessing-classifier combinations spanning seven algorithm families. Combinations were ranked by mean balanced accuracy under 10-fold stratified cross-validation, and the top-ranked model was carried forward to an independent holdout test. **(B)** Cross-validation screen showing, for each algorithm family, the best-performing preprocessing. Bars give mean balanced accuracy (error bars, ± 1 SD across the 10 stratified folds), and diamonds give ROC AUC. LR, logistic regression; KNN, k-nearest neighbors; XGB, extreme gradient boosting; GNB, Gaussian naïve Bayes; GB, gradient boosting; RF, random forest; SVM, support vector machine. **(C)** Predicted probability of inhibition versus serum eculizumab for the selected logistic-regression model (black curve). Observations are shown as rugs at P = 0 (not inhibited, free C5 > 0.5 µg/ml; orange) and P = 1 (inhibited, free C5 ≤ 0.5 µg/ml; blue). The P = 0.5 decision boundary (vertical line) corresponds to *338* µg/ml (shaded band, bootstrap 95% CI *278*–*436* µg/ml). The probability curve was obtained from the unweighted (maximum-likelihood) model; class re-weighting was applied only during model selection **(A, B)**.

Clinical data on serum levels of eculizumab (total drug) and free (unbound) C5 from PNH, aHUS, gMG, and NMOSD were used as inputs (*n* = 390 independent observations) for training and testing of various supervised ML classification algorithms (**Figure 2**, **Table 1**). Observations were labeled as complete inhibition when free C5 concentration was ≤ 0.5 μg/ml (*n* = 87 observations), and incomplete inhibition or non-inhibition when > 0.5 μg/ml (*n* = 303 observations) (**Figure 1B**). Our predictor variable was serum eculizumab concentration, which was measured consistently across the referenced clinical trials. Classifiers were trained on the training subset of patient data, and their relative performance was evaluated using balanced accuracy with 10-fold stratified cross-validation (**Figure 2A**, **Table 1**). The selected and outcompeted models were subsequently evaluated on an independent 20% holdout set that was never used during training or model selection (**Table 1**).

**Table 1:** Classifier performance for predicting C5 inhibition.

Classifier	Preprocessing	Evaluation	Overall						Inhibited (free C5 ≤ 0.5)			Not inhibited		
			Bal. acc.	Acc.	MCC	κ	ROC AUC	PR AUC	Prec.	Recall	F1	Prec.	Recall	F1
LR	log_10_ + z	*CV (n = 312)*	**0.876 ± 0.069**	**0.862**	**0.673**	**0.655**	**0.917**	**0.715**	**0.636**	**0.900**	**0.746**	**0.967**	**0.851**	**0.905**
		*Hold-out (n = 78)*	**0.943**	**0.910**	**0.792**	**0.771**	**0.977**	**0.887**	**0.708**	**1.000**	**0.829**	**1.000**	**0.885**	**0.939**
KNN	robust	*CV (n = 312)*	0.843 ± 0.094	0.891	0.687	0.687	0.885	0.696	0.757	0.757	0.757	0.930	0.930	0.930
		*Hold-out (n = 78)*	0.967	0.949	0.870	0.861	0.962	0.784	0.809	1.000	0.895	1.000	0.934	0.966
XGB	z-score	*CV (n = 312)*	0.823 ± 0.088	0.875	0.643	0.643	0.895	0.676	0.718	0.729	0.723	0.921	0.917	0.919
		*Hold-out (n = 78)*	0.946	0.949	0.858	0.856	0.968	0.790	0.842	0.941	0.889	0.983	0.951	0.967
GNB	log_10_	*CV (n = 312)*	0.807 ± 0.052	0.849	0.588	0.586	0.889	0.646	0.646	0.729	0.685	0.918	0.884	0.901
		*Hold-out (n = 78)*	0.871	0.897	0.713	0.711	0.935	0.808	0.737	0.824	0.778	0.949	0.918	0.933
GB	z-score	*CV (n = 312)*	0.794 ± 0.087	0.853	0.581	0.581	0.887	0.653	0.667	0.686	0.676	0.908	0.901	0.905
		*Hold-out (n = 78)*	0.908	0.923	0.786	0.783	0.972	0.821	0.789	0.882	0.833	0.966	0.934	0.950
RF	robust	*CV (n = 312)*	0.782 ± 0.068	0.843	0.556	0.556	0.875	0.692	0.644	0.671	0.657	0.904	0.893	0.898
		*Hold-out (n = 78)*	0.892	0.897	0.730	0.723	0.957	0.758	0.714	0.882	0.789	0.965	0.902	0.932
SVM	robust	*CV (n = 312)*	0.741 ± 0.100	0.843	0.521	0.517	0.889	0.667	0.684	0.557	0.614	0.878	0.926	0.901
		*Hold-out (n = 78)*	0.837	0.910	0.724	0.719	0.978	0.899	0.857	0.706	0.774	0.922	0.967	0.944

One block per algorithm family, using the best preprocessing per family from the cross-validation screen (selection by balanced accuracy; logistic regression (LR, shaded, values in boldface, is the deployed family), ranked by cross-validated balanced accuracy. CV: 10-fold stratified cross-validation on the 80% training split (n = 312; 70 inhibited / 242 not); balanced accuracy is mean ± SD across folds, other metrics are pooled out-of-fold aggregates. Hold-out: 20% test set (n = 78; 17 inhibited / 61 not). Precision, recall, and F1 are reported per class; recall for the inhibited class is sensitivity, and recall for the not-inhibited class is specificity. Inhibited = free C5 ≤ 0.5 µg/ml (positive, minority class). κ, Cohen’s kappa; PR AUC, area under the precision–recall curve (average precision).

The real-only benchmark showed that serum eculizumab concentration alone carries substantial information about free-C5 blockade. Across the 31 screened combinations, logistic regression on log_10_-scaled concentration values was the best model on real data (mean balanced accuracy 0.88 ± 0.07) (**Figure 2B**, **Table 1**). This logistic regression model is the parsimonious, mechanistically appropriate choice for a monotonic concentration-effect relationship, and we adopted it for all downstream analyses over more flexible learners. On the fixed real holdout set, the logistic model achieved a balanced accuracy of 0.943, an ROC AUC of 0.977, and a specificity of 0.885 (confusion matrix: 54/61 non-inhibited and 17/17 inhibited correctly classified). K-nearest neighbors ranked closely behind with a mean balanced accuracy of 0.84 ± 0.09. Most tree-based and boosting models (random forest, gradient boosting, XGBoost) performed similarly on aggregate metrics but offered no advantage (**Table 1**); random forest in particular yields a non-monotonic, step-like decision function whose threshold estimate is unstable under resampling and therefore unsuitable here. Support vector machines underperformed on minority-class recall (0.56). With a single feature and 22% positive prevalence, an untuned SVM places its decision boundary to favor the majority class, and the Gaussian likelihood of naïve Bayes is misspecified for the heavy-tailed within-class concentration. We therefore retained the screen as evidence that model complexity is unnecessary, consistent with treating this problem as a dose-response estimation problem.

**Figure 3:**
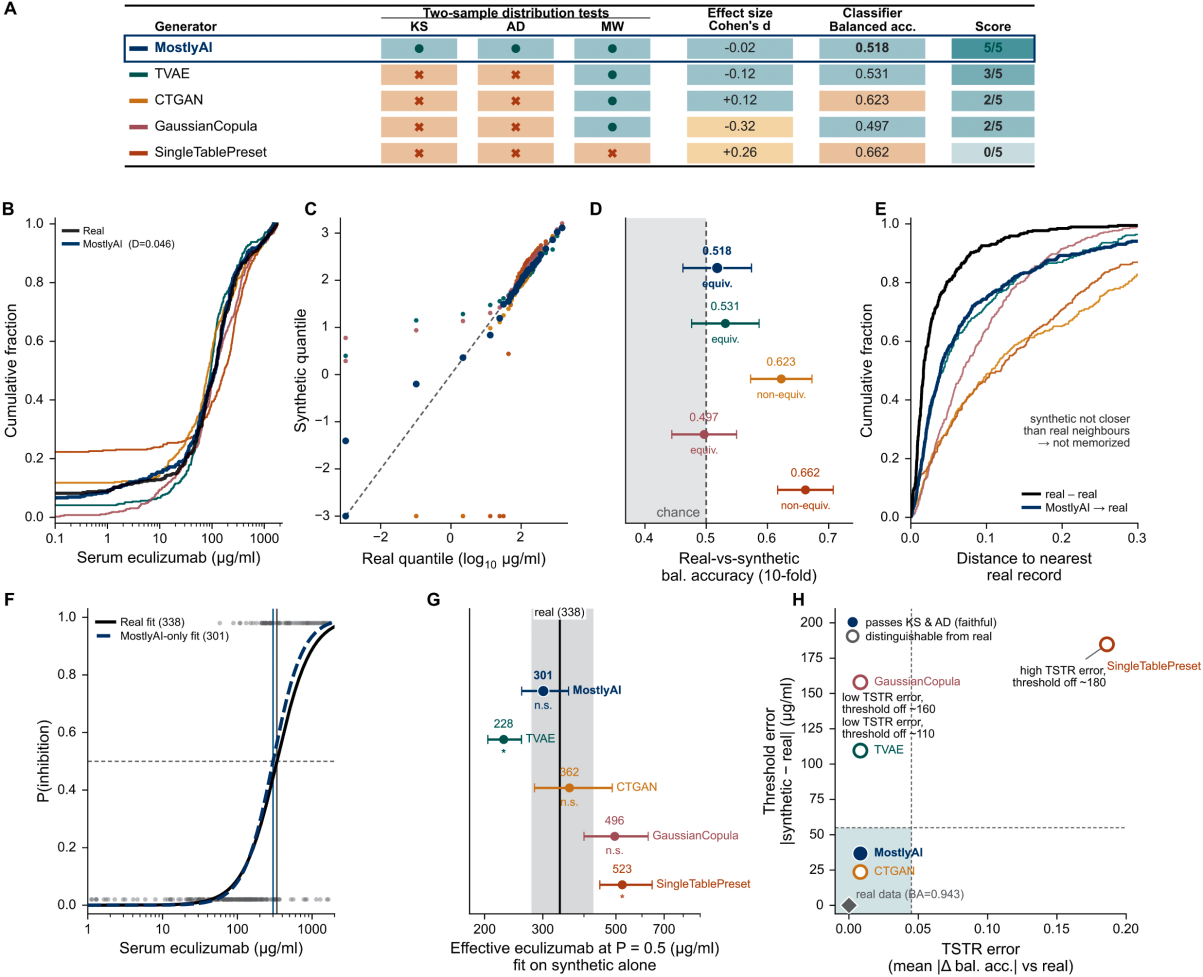
Evaluation and selection of the synthetic data generator. **(A)** Generator scorecard, one row per generator, ranked by the number of criteria met (of five). Filled circle/cross: two-sample test on log_10_[eculizumab] passes (p > 0.05)/fails: KS, AD, MW; effect size: |Cohen’s d| <no><</no> 0.2 (negligible) counts as a pass. Balanced acc.: balanced accuracy of a real-vs-synthetic classifier (10-fold cross-validation), scored as a pass when it cannot exceed chance (mean − SD ≤ 0.50). **(B)** Empirical cumulative distribution and **(C)** quantile-quantile plot of log_10_[eculizumab] for each generator against the real data. **(D)** Real-vs-synthetic discriminator: balanced accuracy (mean ± SD, 10-fold CV) of a classifier trained to separate synthetic from real records. Dashed line and shaded band, chance (0.50); value above each marker, with a TOST verdict below marking equivalence to chance (equiv.) or not (non-equiv.). **(E)** Memorization control: distribution of the distance from each synthetic record to its closest real record, compared with the real-real baseline. **(F)** Recovery of the inhibition-probability curve: logistic P(inhibition) versus serum eculizumab for the real data and for MostlyAI-only training, with the P = 0.5 decision boundary. **(G)** Forest of the P = 0.5 threshold recovered from each generator’s synthetic data alone (point estimate with 95% bootstrap CI); the vertical line is the real-data reference (*338* µg/ml) and the recovered value is printed above each marker. **(H)** Decoupling of statistical fidelity from pharmacological validity: TSTR error (mean |Δ balanced accuracy| vs real) versus threshold-recovery error (|synthetic − real|, µg/ml). Filled markers pass both KS and AD (distributionally faithful); open markers are distinguishable from the real data. The shaded bottom-left corner contains generators that are both faithful and threshold-accurate. Color identifies the generator throughout (key in A).

**Figure 4:**
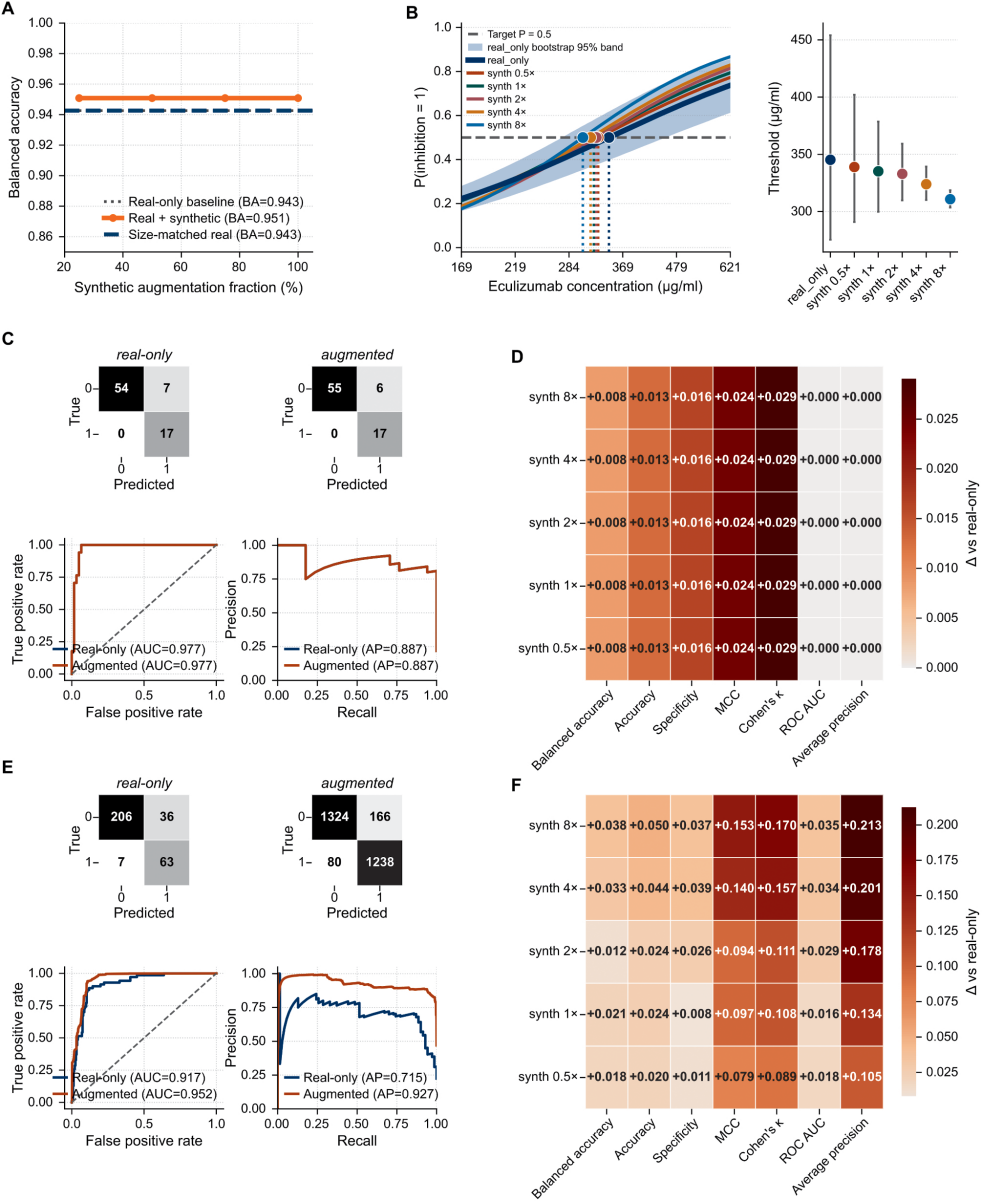
Synthetic−data augmentation of the deployed logistic−regression pipeline: predictive performance and threshold precision as a function of the synthetic−to−real ratio. **(A)** Augmentation learning curve: balanced accuracy on the 20% holdout versus the fraction of the synthetic pool appended to the real training data (orange), with the real−only baseline (dotted) and a size−matched real control (dashed). **(B)** Left: predicted probability of complement inhibition versus serum eculizumab for the real−only model and for each augmentation ratio, with the real−only bootstrap 95% band (shaded) and the P = 0.5 decision threshold (dashed). Right: bootstrap distribution of the P = 0.5 threshold crossing (point, median; whiskers, 95% confidence interval) for each scenario. **(C, E)** Classification diagnostics for the real−only model versus the strongest synthetic augmentation, evaluated on the 20% holdout **(C)** and by 10−fold stratified out−of−fold cross−validation **(E)**: confusion matrices (left, real−only; right, augmented) and the corresponding receiver−operating−characteristic (ROC) and precision-recall (PR) curves. **(D, F)** Change, relative to the real−only model, in seven performance metrics (balanced accuracy, accuracy, specificity, Matthews correlation coefficient [MCC], Cohen’s κ, ROC AUC, and average precision) for each augmentation ratio (rows, 8× top to 0.5× bottom) on the holdout **(D)** and under cross−validation **(F)**; red indicates improvement over real−only, with color centered at zero.

Using the unweighted logistic model, the estimated serum eculizumab concentration associated with the decision boundary for complete C5 inhibition on real data alone was 345.2 µg/ml (95% CI 280–395) (**Figure 2C**). This threshold is very similar to that derived from the previous pharmacologic analysis (**Figure 1D**) and still significantly higher than the 50-100 µg/ml reported in the literature. Three related effective-concentration estimands recur in this work and agree to within their bootstrap confidence intervals: the dose-response crossing on all data (310 µg/ml; **Figure 1D**), the logistic-classifier crossing on all data under cross-validation (338 µg/ml; **Figures 2C**, **3**), and the logistic-classifier crossing on the 80% training split that serves as the augmentation baseline (345.2 µg/ml; **Figure 4**).

### Selection of the synthetic-data generator

Five candidate generators (MostlyAI, CTGAN, TVAE, GaussianCopula, SingleTablePreset) were trained on the *n* = 390 real (serum eculizumab, free-C5) pairs, and asked to synthesize 390 (serum eculizumab, free-C5) pairs matching the real class prevalence (87 inhibited [free C5 ≤ 0.5 µg/ml]/303 not). Synthetic augmentation is only trustworthy if the generator reproduces the real data closely enough to be statistically interchangeable with it, does so without duplicating (memorizing) individual records from the training set, and, for a pharmacological model, without distorting the dose-response relationship that defines the inhibition threshold. We therefore evaluated these generators for descriptive statistics (**Supplementary Table 3**), distributional fidelity to the real data, absence of memorization, and recovery of the pharmacological concentration at which P(inhibition) = 0.5 (inhibition defined as free C5 ≤ 0.5 µg/ml), benchmarked against the real-data logistic reference of 345.2 µg/ml (95% CI 280–395; **Figure 2C**). On the log_10_ scale, the real data had a mean of 1.64 and a standard deviation of 1.50; the MostlyAI generator produced the closest match in log_10_ mean (Δ = 0.03) and log_10_ standard deviation (Δ = 0.17), while SingleTablePreset showed the largest deviation, with a log_10_ mean shift of 0.51 (**Supplementary Table 3**).

MostlyAI was the only generator to satisfy all five fidelity criteria (**Figure 3**, score 5/5; remaining generators 0-3/5). The marginal distribution of serum eculizumab from MostlyAI was indistinguishable from the real data by the Kolmogorov-Smirnov, Anderson-Darling, and Mann-Whitney tests (all p > 0.05) and by effect size (Cohen’s *d* = –0.02, negligible), overlapping the real distribution across the full concentration range (**Figure 3A**). These findings were consistent with the visual assessments, as shown by divergence of the empirical cumulative distribution function (CDF) in the lower or upper quantile range (**Figure 3B**) and from the identity diagonal in the Q-Q plots (**Figure 3C**). In agreement with this, a real-vs-synthetic classifier trained to separate MostlyAI records from real records performed at chance (balanced accuracy 0.518), whereas classifiers trained on the remaining generators separated them all above chance (e.g., the weakest generator, SingleTablePreset, had a balanced accuracy of 0.662) (**Figure 3D**). Furthermore, MostlyAI did not simply duplicate (memorize) the training data, as demonstrated by the fact that the distance from each synthetic record to its nearest real neighbor was no smaller than the real-to-real nearest-neighbor distance (**Figure 3E**).

Statistical fidelity alone is regarded as insufficient to identify a usable generator. We therefore tested whether each generator preserved the concentration-effect relationship by re-deriving the effective concentration (the serum eculizumab at which the modeled probability of inhibition reaches 0.5) from synthetic data alone. The MostlyAI-only logistic fit was close to the real-data fit (P(inhibition) = 0.5 crossing 301 vs 338 µg/ml; **Figure 3F**), with a bootstrap interval contained within the real-data confidence interval, whereas TVAE underestimated (228 µg/ml) and GaussianCopula and SingleTablePreset overestimated (496 and 523 µg/ml) the threshold (**Figure 3G**). The estimates were obtained from all the data under a 10-fold stratified cross-validation.

We then tested the predictive fidelity of the synthetic data generators by carrying out a train-on-synthetic/test-on-real (TSTR) experiment (**Figure 3H**). The performance of TSTR was only weakly discriminating and, in the absence of orthogonal information, might have been misleading: TVAE and GaussianCopula attained TSTR balanced accuracies above the real-trained reference yet recovered an effective concentration ~110-170 µg/ml from the real value, while CTGAN recovered a near-correct threshold despite remaining distributionally distinguishable from real (**Figures 3A, G, H**). MostlyAI was thus the only generator that was simultaneously indistinguishable from the real data, non-memorizing, and, most decisively, faithful to the concentration-effect threshold, and was selected for all subsequent augmentation analyses.

### Augmentation improves classification and sharpens the effective-concentration estimate

The predictive ability of ML models depends on the quantity and quality of the data used to train and validate them. To address the limitations of sample size and class imbalance inherent in our dataset, which are commonplace in rare disease datasets, we asked whether augmenting the real dataset with the MostlyAI synthetic dataset would enhance the prediction performance of our logistic regression pipeline and whether augmentation would affect the precision of the estimated threshold on eculizumab serum concentration (**Figure 4**). To answer these questions, we fixed the ML classifier chosen on real data (logistic regression with log_10_-scaling), let synthetic data enter only within training folds, and computed all reported metrics either on a holdout set of real data that the synthetic generator never informed or through out-of-fold averaging from a 10-fold stratified cross-validation.

First, we asked whether augmentation might raise prediction accuracy. The augmentation learning curve supported a modest benefit beyond simple resampling of the real dataset. With 25%, 50%, 75%, and 100% of the matched MostlyAI synthetic dataset added to the real training data, augmented performance on an exclusively real holdout set was at least as good as the corresponding real-only subsampling control at each fraction (**Figure 4A**). The benefit is not limited to a direct effect of the sample size; in the size-matched control, the augmented learning curve lies above the same-size curve at every fraction, indicating an informational contribution from the synthetic samples beyond that provided by reweighting the real data (**Figure 4A**). Augmentation correctly reclassified a single additional non-inhibited patient on the 78-patient holdout set, yielding a 1.3% increase in accuracy and the smallest increment the test set can resolve (**Figure 4C**). Holdout balanced accuracy rose from 0.943 (real only) to 0.951 and then plateaued, with little further gain beyond a synthetic−to−real ratio of 0.5–1*×* (**Figure 4A**). The same pattern held across the full metric panel (except for AUC ROC and accuracy in the holdout scenario) and under both evaluation schemes (**Figures 4C-F**). Relative to the real−only model, augmentation increased balanced accuracy, MCC, Cohen’s κ, and average precision at every ratio, on both the holdout (**Figure 4D**) and under cross−validation (**Figure 4F**). Under cross-validation, the benefit increased monotonically from 0.5× to 8× (**Figure 4F**). Discrimination on the holdout was identical for the two models (ROC AUC 0.977, average precision 0.887; **Figure 4C**), confirming that the reclassified patient reflects a threshold shift rather than a change in ranking, whereas under cross-validation augmentation improved the mean ROC AUC (from 0.917 to 0.952) and average precision (from 0.715 to 0.927; **Figure 4E**), consistent with reduced variance in the sparse inhibited class.

Second, we investigated the effect of augmentation on the precision of the effective-concentration estimate. As shown in **Figure 4B**, augmentation sharpened the effective−concentration estimate without altering the dose-response relationship. The real-only and augmented probability curves remained closely superimposed at low-to-moderate augmentation, and the P(inhibition) = 0.5 crossing stayed within the real-only 95% CI (275.4–454.0 µg/ml) across all synthetic-to-real ratios (**Figure 4B**, left). Increasing the ratio narrowed the bootstrap 95% CI monotonically from 178.6 µg/ml (real-only) to 15.1 µg/ml at 8× (a 92% reduction), while the point estimate drifted modestly from 345.2 to 310.8 µg/ml, indicating that augmentation sharpened the effective-concentration estimate without displacing it beyond the real-data sampling error (**Figure 4B**, left). Because a procedure that amplified noise would widen this interval, these results argue that faithful augmentation provides genuine benefits. We therefore report an augmentation-stabilized estimate of approximately 310.8–335.2 µg/ml, which is consistent with the pharmacological derivation (**Figures 1C-E**).

Together, these results suggest that conventional serum eculizumab targets may be lower than the concentration required to suppress free C5 below the inhibition cut-off in all patients. We emphasize that this directional inference is robust as the effective concentration is strongly endpoint-dependent, so we present it as hypothesis-generating rather than as a dosing recommendation. The direction is consistent with previous reports that place effective thresholds above conventional targets. The fact that our estimate exceeds previous recommendations follows from our stringent inhibition endpoint, since driving free-C5 to a lower cut-off (free-C5 ≤ 0.5 µg/ml, ~99% suppression of the ~74 µg/ml baseline) necessarily requires more drug. A second contributing factor is that our pooled dataset is covariate- and timing-agnostic, unlike pharmacologic studies on single indications that use steady-state measurements. The magnitude of our estimated target (310.8–335.2 µg/ml) is nonetheless mechanistically plausible, because near-stoichiometric neutralization of a target as abundant as C5 (~0.4 µM) requires a large molar excess of eculizumab, i.e., serum concentrations on the order of hundreds of µg/ml. For example, Reiss et al. (24) linked eculizumab concentrations above 124 µg/ml to complete blockade, while Peffault de Latour et al. (25) observed no episodes of hemolysis in patients with eculizumab concentrations above 150 µg/ml. Clinical trial data in aHUS (and gMG) also report mean serum eculizumab concentrations above 116–150 µg/ml during effective treatment (26). However, substantial inter-patient variability complicates dosing. In aHUS patients and in up to 16% of PNH patients, subtherapeutic eculizumab trough levels have been reported with standard dosing regimens (25, 27–29), whereas other patients exhibit concentrations > 500 µg/ml despite guideline-based dosing (8, 30, 31). This variability underscores the importance of monitoring trough levels and adjusting both dose and interval to avoid treatment failure or overexposure. Importantly, any upward revision of the maintenance concentration target for eculizumab must be weighed against real adverse effects. Higher sustained exposure may increase the already elevated risk of invasive meningococcal and other encapsulated-organism infections; hepatotoxicity has been reported with eculizumab and could plausibly scale with exposure; and, given the drug’s exceptional cost, higher targets carry a substantial financial burden. Conversely, persistent underdosing risks breakthrough hemolysis and disease recurrence. We therefore frame our estimate as a hypothesis to be evaluated against these competing risks in prospective studies, not as a dosing recommendation.

### Limitations

Our study has important limitations. Our dataset suffers from the inherent “small-*n*” barrier that pervades the field of rare diseases, constraining the robustness of meta-analysis. The data were digitized from published figures and therefore lack patient-level covariates (indication, age, weight, renal function, genotype, dosing history, and longitudinal sampling); we cannot stratify or adjust for them, and our estimate is a pooled, population-level, covariate-agnostic concentration-effect threshold rather than an individualized target. We have no independent external real-world cohort, so the estimate is internally validated (held-out real test set, bootstrap intervals, size-matched control, out-of-fold evaluation) but not externally confirmed. The analysis is a steady-state concentration-effect (PD) classifier, not a kinetic model: it yields a concentration target, not a dosing regimen, and does not integrate the time course of exposure, the relationship between free C5 and complement activation biomarkers (e.g., CH50/AH50, sC5b9), or patient-specific genetic factors, or mechanistic PK-PD principles. Synthetic augmentation, while deliberately applied to overcome sample scarcity and demonstrably faithful here, cannot create biological variability absent from the source data (especially in underrepresented groups) and is a precision aid, not a substitute for real evidence. These limitations make prospective validation, ideally integrating mechanistic complement models such as the C-model (32) and primary, covariate-resolved PK-PD data, if or when available, a prerequisite for clinical application.

### The role of faithful synthetic augmentation in rare-disease modeling

Within these limits, the study offers a transferable methodological template for rare-disease modeling. In data-scarce settings, rigorously screened, distributionally faithful synthetic data can improve the predictive performance of ML classifiers and the precision of a simple, interpretable model without inflating its apparent accuracy or substituting for real evidence. Coupled with honest internal validation and explicit uncertainty quantification, this approach can support model-informed pharmacology for rare diseases where conventional data collection is difficult.

## Data Availability

The original contributions presented in the study are included in the article/supplementary material. Further inquiries can be directed to the corresponding authors.
