# Supplementary material for: Data-augmented machine learning refines the effective-concentration estimate for eculizumab in complement-mediated diseases
Source: Front Immunol. 2026 Jul 24;17:1800803. doi: 10.3389/fimmu.2026.1800803 (PMC13447158; doi:10.3389/fimmu.2026.1800803)
Supplement: Supplementary file 1 [file DataSheet1.pdf]

Supplementary Information

Data-augmented machine learning refines the effective-concentration estimate for eculizumab in complement-mediated diseases

Francisco J. Fernández[1,2*#], Lucía Alfonso-González[1,2], Manuel Praga[3], Kaare Mikkelsen[4], M. Cristina Vega[1*#]

[1]Centro de Investigaciones Biológicas Margarita Salas (CIB-CSIC), Consejo Superior de Investigaciones Científicas, 28040 Madrid, Spain.

[2]Abvance Biotech SL, Pharmacokinetics, Pharmacodynamics & Drug Metabolism (PPDM), 28003 Madrid, Spain.

[3]Department of Medicine, Complutense University, 28040 Madrid, Spain.

[4]Aarhus University, Department of Electrical and Computer Engineering, 8000 Aarhus, Denmark.

*** Correspondence:**
Francisco J. Fernández
fjfernandez@abvance.com
M. Cristina Vega
cvega@cib.csic.es

[#]Equally contributing senior authors

Table of contents

Supplementary Table S1. Eculizumab vs free-C5 data (pp. 2–4).

Supplementary Table S2. TRIPOD-AI checklist (pp. 5–6)

Supplementary Table S3. Performance and predictions of ML classifiers (p. 7)

References (p. 8)

Supplementary Table S1. Eculizumab vs free-C5 data used for the model

Source	Disease	Observation 1			Observation 2			Observation 3		
		ID	Eculizumab (µg/mL)	Free C5 (µg/mL)	ID	Eculizumab (µg/mL)	Free C5 (µg/mL)	ID	Eculizumab (µg/mL)	Free C5 (µg/mL)
C08-002A/B; C08-003A/B; NCT00844545; NCT00838513	aHUS	1	5.882	93.529	2	10.78	76.588	3	11.765	121.059
C08-002A/B; C08-003A/B; NCT00844545; NCT00838513	aHUS	4	12.745	100.941	5	12.745	97.412	6	12.745	66
C08-002A/B; C08-003A/B; NCT00844545; NCT00838513	aHUS	7	13.725	95.294	8	20.639	41.538	9	21.569	53.647
C08-002A/B; C08-003A/B; NCT00844545; NCT00838513	aHUS	10	21.569	41.647	11	22.549	54	12	23.529	60
C08-002A/B; C08-003A/B; NCT00844545; NCT00838513	aHUS	13	24.51	44.118	14	24.51	40.235	15	24.57	43.314
C08-002A/B; C08-003A/B; NCT00844545; NCT00838513	aHUS	16	27.451	48.653	17	27.451	33.529	18	29.412	40.235
C08-002A/B; C08-003A/B; NCT00844545; NCT00838513	aHUS	19	30.392	52.941	20	30.392	38.824	21	30.392	33.529
C08-002A/B; C08-003A/B; NCT00844545; NCT00838513	aHUS	22	31.373	42.706	23	31.373	40.931	24	31.373	40.235
C08-002A/B; C08-003A/B; NCT00844545; NCT00838513	aHUS	25	31.373	39.529	26	32.432	42.249	27	34.314	48.706
C08-002A/B; C08-003A/B; NCT00844545; NCT00838513	aHUS	28	34.314	43.412	29	35.294	35.294	30	35.294	28.235
C08-002A/B; C08-003A/B; NCT00844545; NCT00838513	aHUS	31	36.275	34.235	32	40.196	17.647	33	41.176	35.294
C08-002A/B; C08-003A/B; NCT00844545; NCT00838513	aHUS	34	41.176	22.941	35	43.137	28.588	36	43.26	27.692
C08-002A/B; C08-003A/B; NCT00844545; NCT00838513	aHUS	37	44.226	24.142	38	45.098	33.529	39	45.098	26.824
C08-002A/B; C08-003A/B; NCT00844545; NCT00838513	aHUS	40	46.078	35.647	41	46.078	34.588	42	46.078	28.941
C08-002A/B; C08-003A/B; NCT00844545; NCT00838513	aHUS	43	46.078	27.176	44	46.078	23.294	45	46.078	18
C08-002A/B; C08-003A/B; NCT00844545; NCT00838513	aHUS	46	46.192	28.402	47	50	33.176	48	50.123	29.822
C08-002A/B; C08-003A/B; NCT00844545; NCT00838513	aHUS	49	50.98	46.941	50	50.98	24.706	51	50.98	23.697
C08-002A/B; C08-003A/B; NCT00844545; NCT00838513	aHUS	52	51.106	29.822	53	51.106	23.787	54	51.123	27.692
C08-002A/B; C08-003A/B; NCT00844545; NCT00838513	aHUS	55	51.961	29.647	56	52.941	28.235	57	53.071	12.426
C08-002A/B; C08-003A/B; NCT00844545; NCT00838513	aHUS	58	53.922	12.706	59	54.037	24.852	60	54.054	26.627
C08-002A/B; C08-003A/B; NCT00844545; NCT00838513	aHUS	61	54.902	19.412	62	55.037	11.361	63	55.037	14.201
C08-002A/B; C08-003A/B; NCT00844545; NCT00838513	aHUS	64	56.863	31.059	65	57.002	30.533	66	59	31.059
C08-002A/B; C08-003A/B; NCT00844545; NCT00838513	aHUS	67	63.725	16.235	68	64.706	27.176	69	64.706	27.176
C08-002A/B; C08-003A/B; NCT00844545; NCT00838513	aHUS	70	64.706	21.882	71	64.706	12.706	72	64.865	14.201
C08-002A/B; C08-003A/B; NCT00844545; NCT00838513	aHUS	73	64.865	12.071	74	65.686	23.297	75	65.848	14.911
C08-002A/B; C08-003A/B; NCT00844545; NCT00838513	aHUS	76	66.83	14.201	77	66.83	13.136	78	66.83	12.071
C08-002A/B; C08-003A/B; NCT00844545; NCT00838513	aHUS	79	68.796	19.172	80	69.608	13.412	81	69.779	18.462
C08-002A/B; C08-003A/B; NCT00844545; NCT00838513	aHUS	82	70.588	18.7	83	70.588	11.294	84	70.762	20.947
C08-002A/B; C08-003A/B; NCT00844545; NCT00838513	aHUS	85	70.762	14.911	86	70.762	18.817	87	71.744	16.686
C08-002A/B; C08-003A/B; NCT00844545; NCT00838513	aHUS	88	72.549	14.471	89	73.71	20.592	90	74.51	11.294
C08-002A/B; C08-003A/B; NCT00844545; NCT00838513	aHUS	91	74.693	25.917	92	74.693	17.751	93	74.693	10.651
C08-002A/B; C08-003A/B; NCT00844545; NCT00838513	aHUS	94	74.693	19.527	95	75.49	18.353	96	75.49	15.529
C08-002A/B; C08-003A/B; NCT00844545; NCT00838513	aHUS	97	76.471	24	98	76.471	9.882	99	78.431	11.647
C08-002A/B; C08-003A/B; NCT00844545; NCT00838513	aHUS	100	78.624	23.787	101	78.624	9.586	102	79	26.118
C08-002A/B; C08-003A/B; NCT00844545; NCT00838513	aHUS	103	80.392	24	104	80.392	9.176	105	80.59	7.811
C08-002A/B; C08-003A/B; NCT00844545; NCT00838513	aHUS	106	81.373	13.0559	107	81.572	13.846	108	83.333	14.824
C08-002A/B; C08-003A/B; NCT00844545; NCT00838513	aHUS	109	83.538	22.367	110	83.538	16.686	111	84.314	23.294
C08-002A/B; C08-003A/B; NCT00844545; NCT00838513	aHUS	112	84.314	16.941	113	84.521	13.846	114	86.486	7.811
C08-002A/B; C08-003A/B; NCT00844545; NCT00838513	aHUS	115	88.235	11.294	116	89.435	15.621	117	90.418	15.266
C08-002A/B; C08-003A/B; NCT00844545; NCT00838513	aHUS	118	91.4	7.811	119	95.332	6.746	120	96.314	5.68
C08-002A/B; C08-003A/B; NCT00844545; NCT00838513	aHUS	121	96.314	8.521	122	96.314	13.136	123	99.02	8.471
C08-002A/B; C08-003A/B; NCT00844545; NCT00838513	aHUS	124	100.246	9.231	125	101.229	8.166	126	102.2941	9.529

Source	Disease	Observation 1			Observation 2			Observation 3		
		ID	Eculizumab (µg/mL)	Free C5 (µg/mL)	ID	Eculizumab (µg/mL)	Free C5 (µg/mL)	ID	Eculizumab (µg/mL)	Free C5 (µg/mL)
C08-002A/B; C08-003A/B; NCT00844545; NCT00838513	aHUS	127	104.177	8.876	128	104.177	13.136	129	105.106	8.521
C08-002A/B; C08-003A/B; NCT00844545; NCT00838513	aHUS	130	105.882	8.471	131	107.843	14.118	132	108.824	15.88
C08-002A/B; C08-003A/B; NCT00844545; NCT00838513	aHUS	133	109.091	9.586	134	109.091	8.166	135	109.091	13.846
C08-002A/B; C08-003A/B; NCT00844545; NCT00838513	aHUS	136	110.074	7.811	137	110.784	10.941	138	112.039	19.172
C08-002A/B; C08-003A/B; NCT00844545; NCT00838513	aHUS	139	114.005	7.811	140	115.972	17.751	141	118.627	9.882
C08-002A/B; C08-003A/B; NCT00844545; NCT00838513	aHUS	142	118.919	6.036	143	118.919	12.071	144	122.85	13.136
C08-002A/B; C08-003A/B; NCT00844545; NCT00838513	aHUS	145	123.833	11.361	146	124.816	7.101	147	124.816	3.55
C08-002A/B; C08-003A/B; NCT00844545; NCT00838513	aHUS	148	124.816	4.615	149	124.816	8.521	150	125.799	4.97
C08-002A/B; C08-003A/B; NCT00844545; NCT00838513	aHUS	151	125.799	17.396	152	127.764	6.746	153	127.764	13.846
C08-002A/B; C08-003A/B; NCT00844545; NCT00838513	aHUS	154	128.431	5.294	155	128.747	6.746	156	129.412	7.765
C08-002A/B; C08-003A/B; NCT00844545; NCT00838513	aHUS	157	129.73	7.101	158	129.73	6.746	159	130.713	11.361
C08-002A/B; C08-003A/B; NCT00844545; NCT00838513	aHUS	160	130.716	5.68	161	131.695	9.231	162	131.695	20.237
C08-002A/B; C08-003A/B; NCT00844545; NCT00838513	aHUS	163	132.353	18	164	132.678	5.68	165	133.661	4.615
C08-002A/B; C08-003A/B; NCT00844545; NCT00838513	aHUS	166	133.661	5.325	167	135.627	17.751	168	137.592	12.426
C08-002A/B; C08-003A/B; NCT00844545; NCT00838513	aHUS	169	139	12.7	170	139.216	11.294	171	140.196	11.294
C08-002A/B; C08-003A/B; NCT00844545; NCT00838513	aHUS	172	141.523	4.615	173	142.506	16.686	174	143.137	9.882
C08-002A/B; C08-003A/B; NCT00844545; NCT00838513	aHUS	175	144.472	7.101	176	145.455	5.325	177	147.059	14.118
C08-002A/B; C08-003A/B; NCT00844545; NCT00838513	aHUS	178	147.42	7.811	179	148.039	9.176	180	148.403	7.811
C08-002A/B; C08-003A/B; NCT00844545; NCT00838513	aHUS	181	149.02	8.118	182	149.386	7.811	183	149.386	12.781
C08-002A/B; C08-003A/B; NCT00844545; NCT00838513	aHUS	184	150	8.824	185	150	8.118	186	150.369	3.55
C08-002A/B; C08-003A/B; NCT00844545; NCT00838513	aHUS	187	150.369	8.166	188	150.369	8.876	189	150.369	14.201
C08-002A/B; C08-003A/B; NCT00844545; NCT00838513	aHUS	190	152.334	9.231	191	153.317	6.036	192	153.317	10.296
C08-002A/B; C08-003A/B; NCT00844545; NCT00838513	aHUS	193	154.283	8.521	194	154.902	8.824	195	159.214	6.036
C08-002A/B; C08-003A/B; NCT00844545; NCT00838513	aHUS	196	159.214	9.231	197	160.784	7.059	198	162.162	8.166
C08-002A/B; C08-003A/B; NCT00844545; NCT00838513	aHUS	199	164.128	8.521	200	168.627	9.529	201	170.025	9.941
C08-002A/B; C08-003A/B; NCT00844545; NCT00838513	aHUS	202	171.007	9.941	203	174	9.529	204	174.939	9.231
C08-002A/B; C08-003A/B; NCT00844545; NCT00838513	aHUS	205	176.904	9.586	206	179.853	8.876	207	179.853	6.036
C08-002A/B; C08-003A/B; NCT00844545; NCT00838513	aHUS	208	181	7.765	209	181.818	8.521	210	189.681	6.036
C08-002A/B; C08-003A/B; NCT00844545; NCT00838513	aHUS	211	193.612	7.101	212	194.595	10.296	213	200.491	4.26
C08-002A/B; C08-003A/B; NCT00844545; NCT00838513	aHUS	214	206.388	6.391	215	206.863	7.059	216	209.337	5.68
C08-002A/B; C08-003A/B; NCT00844545; NCT00838513	aHUS	217	210.319	10.296	218	211.765	8.118	219	213.268	6.391
C08-002A/B; C08-003A/B; NCT00844545; NCT00838513	aHUS	220	219.165	7.101	221	220.588	7.412	222	227	91.598
C08-002A/B; C08-003A/B; NCT00844545; NCT00838513	aHUS	223	239.803	2.84	224	245.098	9.882	225	245.7	9.231
C08-002A/B; C08-003A/B; NCT00844545; NCT00838513	aHUS	226	249.614	3.55	227	251.961	4.588	228	277.451	7.059
C08-002A/B; C08-003A/B; NCT00844545; NCT00838513	aHUS	229	288.235	7.412	230	305.882	6	231	316	7.059
C08-002A/B; C08-003A/B; NCT00844545; NCT00838513	aHUS	232	317	7	233	329	4.235			
Ref. 1	gMG	234	0.08722747	120	235	328.7	2.39	236	124.8	1
Ref. 1	gMG	237	453.85	0.49	238	367.57	0.68	239	843.13	0.017
Ref. 1	gMG	240	356.18	3.62	241	844.94	0.65	242	322.74	1.74
Ref. 1	gMG	243	775.43	0.54	244	363.23	1.55	245	874.74	0.12
Ref. 1	gMG	246	317.39	1.1	247	765.91	0.021			
NCT01892345	NMOSD	248	4.06071654	297.275774	249	0	255.906383	250	0	252.148263
NCT01892345	NMOSD	251	1.12111087	214.721892	252	2.29518761	208.496941	253	1.12993852	198.626613
NCT01892345	NMOSD	254	0.3354505	192.739893	255	1.87146067	188.225638	256	2.97491625	185.01921
NCT01892345	NMOSD	257	0	182.42761	258	3.16912443	178.060674	259	1.64194191	177.382709
NCT01892345	NMOSD	260	0	172.169444	261	4.6256858	167.211731	262	8.03315663	161.733895
NCT01892345	NMOSD	263	0	157.359443	264	1.30649141	154.047787	265	1.30649141	150.614368
NCT01892345	NMOSD	266	1.28000847	147.843881	267	0.40607165	143.830208	268	0	140.444893
NCT01892345	NMOSD	269	0	137.190361	270	17.4963917	134.510069	271	0	133.584069
NCT01892345	NMOSD	272	0.67972864	130.555023	273	0	127.449313	274	0	124.713401

Source	Disease	Observation 1			Observation 2			Observation 3		
		ID	Eculizumab (μg/mL)	Free C5 (μg/mL)	ID	Eculizumab (μg/mL)	Free C5 (μg/mL)	ID	Eculizumab (μg/mL)	Free C5 (μg/mL)
NCT01892345	NMOSD	275	0	121.573115	276	0	117.577481	277	0	114.515365
NCT01892345	NMOSD	278	1.74787364	108.53845	279	0	106.205409	280	0	104.163998
NCT01892345	NMOSD	281	0	101.864028	282	0	100.260062	283	0	98.6560961
NCT01892345	NMOSD	284	0	96.614685	285	0	94.4274588	286	0	92.6776779
NCT01892345	NMOSD	287	0	91.2195271	288	0	89.3254344	289	0	86.700763
NCT01892345	NMOSD	290	22.8812549	84.9765372	291	0	80.7028025	292	22.854772	76.4531198
NCT01892345	NMOSD	293	0	72.1312812	294	0	69.0691645	295	0	62.9569572
NCT01892345	NMOSD	296	16.3134873	63.2546004	297	31.5058638	57.4896435	298	58.3065929	0.12234477
NCT01892345	NMOSD	299	88.7178288	0.26515335	300	109.736451	0.12384801	301	131.258248	0.17195196
NCT01892345	NMOSD	302	149.902234	0.17044871	303	179.757328	0.31810696	304	204.580665	0.16593896
NCT01892345	NMOSD	305	247.809641	0.16443572	306	265.791553	0.31025079	307	283.773465	0.30874754
NCT01892345	NMOSD	308	343.209996	0.01260764	309	374.309789	0.01260764	310	413.58398	0.15842272
NCT01892345	NMOSD	311	448.929869	0.26665659	312	471.661054	0.26665659	313	496.63446	0.37639372
NCT01892345	NMOSD	314	523.92071	0.57782898	315	561.561787	0.57632573	316	596.748778	0.4290074
NCT01892345	NMOSD	317	631.741562	0.4290074	318	672.207485	0.22606889	319	707.00606	0.37188397
NCT01892345	NMOSD	320	742.193052	0.03515637	321	792.316418	0.18097145	322	816.82196	0.76423176
NCT01892345	NMOSD	323	842.121989	0.26665659	324	864.058686	0.26665659	325	906.687382	0.2606436
NCT01892345	NMOSD	326	929.886432	0.40645868	327	964.746801	0.20051367	328	1001.45215	0.45606587
NCT01892345	NMOSD	329	1042.71256	0.3042378	330	1081.98675	0.15691947	331	1113.934	0.2636501
NCT01892345	NMOSD	332	1142.27074	0.56730624	333	1174.63288	0.17379514	334	1215.42543	0.2636501
NCT01892345	NMOSD	335	1244.23003	0.46508536	336	1289.25985	0.61090044	337	1329.34618	0.2200559
NCT01892345	NMOSD	338	1361.96433	0.22189908	339	1395.35048	0.22189908	340	1426.25606	0.16928539
NCT01892345	NMOSD	341	1464.40032	0.17078864	342	1507.35563	0.26665659	343	1547.01824	0.26665659
NCT02003144	NMOSD	344	1586.48664	0.61725336	345	1606.18112	0.47444478	346	1650.54003	0.8833283
NCT02003144	NMOSD	347	1692.36541	0.15425291	348	1717.27702	0.30157123	349	1755.64197	0.09829279
Ref. 2	PNH	350	0	0.052	351	216	0.052	352	108	0.046
Ref. 2	PNH	353	162	0.044	354	310	0.041	355	216	0.046
Ref. 2	PNH	356	229	0.035	357	229	0.044	358	229	0.018
Ref. 2	PNH	359	472	0.052	360	229	0.046	361	229	0.046
Ref. 2	PNH	362	243	0.059	363	243	0.049	364	445	0.066
Ref. 2	PNH	365	216	0.049	366	216	0.059	367	229	0.079
Ref. 2	PNH	368	243	0.044	369	367	0.052	370	489	0.052
Ref. 2	PNH	371	353	0.046	372	285	0.044	373	557	0.041
Ref. 2	PNH	374	367	0.046	375	285	0.035	376	272	0.044
Ref. 2	PNH	377	272	0.018	378	272	0.052	379	503	0.046
Ref. 2	PNH	380	272	0.046	381	272	0.059	382	244	0.049
Ref. 2	PNH	383	272	0.066	384	517	0.049	385	244	0.059
Ref. 2	PNH	386	244	0.079	387	299	0.044	388	285	0.066
Ref. 3	PNH	389	0	140						
Ref. 4	PNH	390	0	316.76						

Supplementary Table S2. TRIPOD-AI checklist.

Section/Topic		Checklist Item	Page
**Title and abstract**			
Title	1	Identify the study as developing and/or validating a multivariable prediction model, the target population, and the outcome to be predicted.	1
Abstract	2	Provide a summary of objectives, study design, setting, participants, sample size, predictors, outcome, statistical analysis, results, and conclusions.	2
**Introduction**			
Background and objectives	3a	Explain the medical context (including whether diagnostic or prognostic) and rationale for developing or validating the multivariable prediction model, including references to existing models.	3-4
	3b	Specify the objectives, including whether the study describes the development or validation of the model or both.	4
**Methods**			
Source of data	4a	Describe the study design or source of data (e.g., randomized trial, cohort, or registry data), separately for the development and validation data sets, if applicable.	5
	4b	Specify the key study dates, including start of accrual; end of accrual; and, if applicable, end of follow-up.	5; Table S1
Participants	5a	Specify key elements of the study setting (e.g., primary care, secondary care, general population) including number and location of centres.	5; Table S1
	5b	Describe eligibility criteria for participants.	5
	5c	Give details of treatments received, if relevant.	5
Outcome	6a	Clearly define the outcome that is predicted by the prediction model, including how and when assessed.	5
	6b	Report any actions to blind assessment of the outcome to be predicted.	NA
Predictors	7a	Clearly define all predictors used in developing or validating the multivariable prediction model, including how and when they were measured.	5, 7
	7b	Report any actions to blind assessment of predictors for the outcome and other predictors.	NA
Sample size	8	Explain how the study size was arrived at.	5, 7
Missing data	9	Describe how missing data were handled (e.g., complete-case analysis, single imputation, multiple imputation) with details of any imputation method.	5, 9
Statistical analysis methods	10a	Describe how predictors were handled in the analyses.	5, 7
	10b	Specify type of model, all model-building procedures (including any predictor selection), and method for internal validation.	7
	10d	Specify all measures used to assess model performance and, if relevant, to compare multiple models.	7
Risk groups	11	Provide details on how risk groups were created, if done.	NA
**Results**			
Participants	13a	Describe the flow of participants through the study, including the number of participants with and without the outcome and, if applicable, a summary of the follow-up time. A diagram may be helpful.	7, 10
	13b	Describe the characteristics of the participants (basic demographics, clinical features, available predictors), including the number of participants with missing data for predictors and outcome.	Table S1
Model development	14a	Specify the number of participants and outcome events in each analysis.	7, 12
	14b	If done, report the unadjusted association between each candidate predictor and outcome.	NA
Model specification	15a	Present the full prediction model to allow predictions for individuals (i.e., all regression coefficients, and model intercept or baseline survival at a given time point).	14
	15b	Explain how to the use the prediction model.	14-15
Model performance	16	Report performance measures (with CIs) for the prediction model.	13-16; 20-22

**Discussion**			
Limitations	18	Discuss any limitations of the study (such as nonrepresentative sample, few events per predictor, missing data).	23
Interpretation	19b	Give an overall interpretation of the results, considering objectives, limitations, and results from similar studies, and other relevant evidence.	19-23
Implications	20	Discuss the potential clinical use of the model and implications for future research.	20-23
**Other information**			
Supplementary information	21	Provide information about the availability of supplementary resources, such as study protocol, Web calculator, and data sets.	25; SI
Funding	22	Give the source of funding and the role of the funders for the present study.	24

Supplementary Table S3. Descriptive statistics for the real data and the five synthetic generators.

		Raw concentration (µg/ml)							log_{10} concentration			Similarity
Dataset	n	Mean	Median	SD	Q1	Q3	Min.	Max.	Mean	Median	SD	Distance
**Real**	**390**	**224.8**	**111.4**	**338.1**	**46.1**	**229.0**	**0.0**	**1755.6**	**1.640**	**2.047**	**1.498**	**—**
**MostlyAI**	**390**	**208.4**	**116.2**	**308.7**	**39.8**	**216.0**	**0.0**	**1507.4**	**1.674**	**2.065**	**1.319**	**0.183**
CTGAN	390	221.6	84.4	350.5	30.6	235.3	0.0	1755.6	1.446	1.926	1.716	0.316
TVAE	390	183.0	94.6	273.4	50.4	168.3	0.0	1668.8	1.797	1.976	1.107	0.428
GaussianCopula	390	256.1	108.3	360.6	55.2	318.0	0.1	1755.6	2.009	2.035	0.701	0.878
SingleTablePreset	390	268.6	173.2	321.6	13.8	359.2	0.0	1706.9	1.137	2.239	2.265	0.938

Rows show descriptive statistics for serum eculizumab concentrations in the real dataset and each synthetic generator. Synthetic rows are ranked by proximity distance, defined as Euclidean distance from the real row across log10 mean, log10 SD and log10 median; smaller values indicate closer agreement in the log-scale summary space. The nearest synthetic generator is shaded blue.

References

1. Monteleone, J. P. R., Gao, X., Kleijn, H. J., Bellanti, F. & Pelto, R. Eculizumab Pharmacokinetics and Pharmacodynamics in Patients With Generalized Myasthenia Gravis. *Front Neurol* **12**, 696385 (2021).
2. Peffault de Latour, R. *et al.* Pharmacokinetic and pharmacodynamic effects of ravulizumab and eculizumab on complement component 5 in adults with paroxysmal nocturnal haemoglobinuria: results of two phase 3 randomised, multicentre studies. *British Journal of Haematology* **191**, 476–485 (2020).
3. Röth, A. *et al.* The SMART Anti-hC5 Antibody (SKY59/RO7112689) Shows Good Safety and Efficacy in Patients with Paroxysmal Nocturnal Hemoglobinuria (PNH). *Blood* **132**, 535 (2018).
4. Röth, A. *et al.* The complement C5 inhibitor crovalimab in paroxysmal nocturnal hemoglobinuria. *Blood* **135**, 912–920 (2020).
